# Microbial Community Structure, Diversity, and Succession During Decomposition of Kiwifruit Litters with Different Qualities

**DOI:** 10.3390/microorganisms12122498

**Published:** 2024-12-04

**Authors:** Yupeng Lu, Zhu Gao, Yulin Zhu, Dongliang Yao, Xiaoling Wang

**Affiliations:** 1 Jiangxi Provincial Key Laboratory of Plantation and High Valued Utilization of Specialty Fruit Tree and Tea, Institute of Biological Resources, Jiangxi Academy of Sciences, Nanchang 330096, China; luyupeng@jxas.ac.cn (Y.L.); gaozhu@jxas.ac.cn (Z.G.); zhuyulin@jxas.ac.cn (Y.Z.); yaodongliang@jxas.ac.cn (D.Y.); 2 Jiangxi Kiwifruit Engineering Research Center, Nanchang 330096, China

**Keywords:** microbial community, litter decomposition, litter quality, variety difference, kiwifruit, orchard ecosystem

## Abstract

There are differences in the litter quality and decomposition rate of kiwifruit varieties, but it is not clear whether these differences are related to microbial communities. The leaf litters of two kiwifruit varieties (*A. chinensis* cv ‘Hongyang’ and *A. chinensis* cv ‘Jinyan’) were taken as objects, and the structure, diversity, and succession of the soil microbial communities were analyzed using an in situ decomposition experiment. Moreover, the contents of C, N, P, and K in the litters during decomposition were analyzed. The results show that there were variety differences in community structure at the generic level. *Lophotrichus*, *Acaulium*, and *Fusarium* were relatively more abundant in the microbial community of the ‘Hongyang’ kiwifruit litter, and *Humicola* and *Tausonia* were relatively more abundant in the microbial community of the ‘Jinyan’ kiwifruit litter. *Subgroup_6* and *Sphingomonas* were the dominant bacteria. The bacterial community diversity of ‘Jinyan’ kiwifruit was higher than that of the ‘Hongyang’ kiwifruit litter. The community diversity was higher in the middle and later periods. The contents of C and N in the litters were the main factors affecting microbial communities. The abundances of *Humicola* and *Apiotrichum* were negatively correlated with the contents of C and N, and the abundances of *Sphingomonas* and *SC-I-84* were positively correlated with the content of C. There were variety differences in the microbial communities corresponding to the decomposition processes of the ‘Hongyang’ and ‘Jinyan’ kiwifruit litters. The mechanisms of the variety differences were related to litter quality and the initial soil microbial community.

## 1. Introduction

Litter decomposition is an important part of nutrient cycling in ecosystems [1,2]. Nutrient elements, such as C, N, P, and K, are leached, degraded, and transformed from plant residues into the soil bank and will be reabsorbed and utilized by plants, thus forming a nutrient cycle. The litter decomposition process includes physical fragmentation, leaching, biodegradation, and other processes. Microorganisms are the main participants in biodegradation and can be responsible for up to 90% of organic matter degradation [3]. Microorganisms mediate nutrient cycling in agroecosystems through organic matter decomposition, nutrient release, and the facilitation of plant uptake [4]. Moreover, microbial diversity drives ecosystem multifunctionality [5]. In other words, microorganisms are one of the engines driving the functioning of ecosystems.

Microorganisms are microscopic organisms, such as bacteria, fungi, actinomyces, archaea, and other individual cells, which play different roles in the biodegradation of litter. Bacteria are a type of R-selected strategy decomposers with a large biomass and a rapid growth rate; they prefer to utilize substrates with low C/N ratios and degrade substances such as cellulose, hemicellulose, and pectin in substrates by releasing extracellular enzymes [6]. *Proteobacteria*, *Bacteroidetes*, *Acidobacteria*, and *Actinobacteria* are the main bacteria found to be involved in litter decomposition, and they generally have the ability to produce proteolytic and cellulolytic enzymes [7]. Fungi are a type of K-selected strategy decomposers with a slow biomass turn-over and are more competitive against substrates with high C/N ratios [8]. Fungi colonize and destroy the surface structure of litter through mycelium and secrete extracellular enzymes to degrade lignocellulose [9]. *Ascomycota* and *Basidiomycota* are the main fungi in litter decomposition, and they can produce extracellular hydrolytic and oxidating enzymes. *Ascomycota* are mainly soft rot fungi, which mainly decompose cellulose and hemicellulose, and they have limited degradation capacity for lignin and are generally found in high abundances in the early decomposition stage [10]. *Basidiomycota* include white and brown rot fungi, which can produce laccase and manganese peroxidase (MnP), and they play an important role in the lignin degradation process during later stages of litter decomposition [11]. Some generic-level microorganisms, such as *Sphingomonas*, *Pseudomonas*, *Penicillium*, *Fusarium*, and *Aspergillus*, have been found in previous studies to be important decomposers in litter decomposition [9,12].

Microbial communities were mainly affected by litter quality and environmental factors such as climate and soil during litter decomposition [13,14,15]. The litter quality is reflected by the initial contents of C, N, P, and lignin. In general, the litter with low C/N and lignin/N ratios is considered to be of higher quality, while the litter with high C/N and lignin/N ratios is considered to be of lower quality [16,17]. As a source of microbial metabolic activity, litter quality significantly affects microbial communities. The C or N limitation of microbial growth is the driving force for its degradation of litter substrates [18,19]. Microorganisms maintain C and N balance between litters and decomposers by adjusting C use efficiency, accumulating N, or adjusting extracellular enzyme allocation between C and N [18]. Bacteria can rapidly use the N sources in litter by nitrate reduction and organic N degradation to grow, multiply, and increase their biomass [20]. Thus, the abundances of bacteria, especially Gram-negative bacteria, are higher in high-quality litter during decomposition [8]. The abundances of fungi and Gram-positive bacteria are higher in low-quality litter. Moreover, along with the changes in litter quality during the decomposition process (a shift from carbohydrates and aliphatic components to aromatic compounds), the microbial community structure undergoes succession [3].

The litter quality is mainly affected by the biological characteristics of plants. For example, the litter quality of broad-leaved species is generally higher than that of coniferous species [21]. Furthermore, even within the same species, there are differences in litter quality among different varieties. Compared with a single-species litter, the mixture of multiple species also significantly affects the quality of litters [22,23]. Therefore, the dominant species in the ecosystem can affect the microbial community through the quality of the litter and the impact on the soil environment. Specifically, the dominant species affect the physicochemical properties of soil (temperature, moisture, and nutrients) through litter deposition and nutrient release, root exudates, and the shaping of understory vegetation, thus affecting the microbial community [15,20].

In the orchard ecosystem, litter mainly consists of the branches and leaves of fruit trees, which occur by natural fall and artificial pruning, and understory herbaceous plant residues [24,25,26]. Litter decomposition is an important supplement to soil fertility in orchards. Kiwifruit (*Actinidia* spp.) is a nutritious fruit and is cultivated in China, New Zealand, Italy, Chile, and other countries [27]. The branches and leaves of kiwifruit trees pruned in winter are the main source of litter in the orchard. Lu et al. [28] reported that there were variety differences in the litter quality and decomposition rate of kiwifruit. However, is the variety difference in litter decomposition related to microbial communities? What is the microbial mechanism for the variety difference in litter decomposition? Here, we propose two hypotheses: (1) there are differences in the microbial communities during litter decomposition between different varieties; (2) the differences in microbial communities are affected by litter quality. This study took the leaf litter of two kiwifruit varieties (*A. chinensis* cv ‘Hongyang’ and *A. chinensis* cv ‘Jinyan’) as objects and analyzed the structure, diversity, and succession of soil microbial communities using an in situ decomposition experiment. The fungal and bacterial communities at the phylum and generic taxonomic level were analyzed by the amplicon sequencing of ITS and 16S rRNA genes, respectively. Moreover, the contents of C, N, P, and K in the the litters during decomposition were analyzed to verify the relationship between litter quality and microbial community.

## 2. Materials and Methods

### 2.1. Study Site and Plant Species

**Figure 1:**
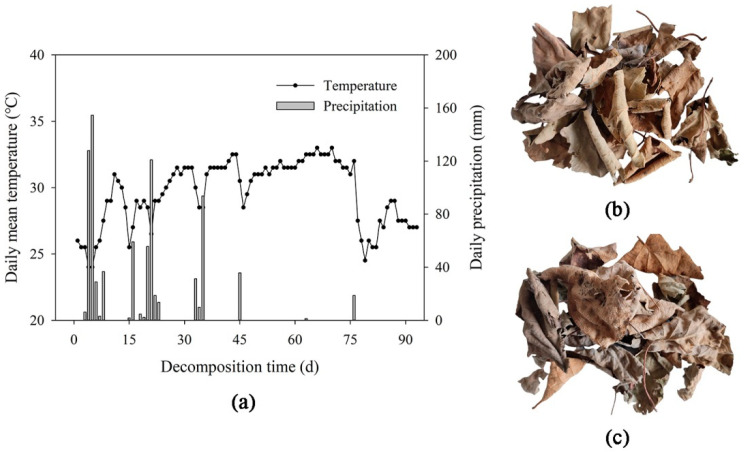
Meteorological conditions of study site and kiwifruit litter. (**a**) shows daily mean temperature and daily precipitation of study site during decomposition experiment. (**b**) shows ‘Hongyang’ kiwifruit litter. (**c**) shows ‘Jinyan’ kiwifruit litter.

The study site was an 11-year-old kiwifruit orchard, which was located in Fengxin County (28°40′36″ N, 115°19′02″ E), Jiangxi Province, Southeast China. The climate type was subtropical monsoon climate, and the soil type was red soil [28]. In order to ensure the activity of microbial community during litter decomposition, we chose the warmer and wetter months of June to September to carry out the decomposition experiment. During the 90-day decomposition experiment, the average temperature in the study site was in the range of 25 to 30 °C, and the total precipitation was 824 mm, providing a suitable environment for microbial decomposition (Figure 1a). More detailed meteorological data are shown in Table S1.

‘Hongyang’ and ‘Jinyan’ kiwifruit are the two main cultivars of *Actinidia chinensis*, which are widely cultivated in Southern China. ‘Hongyang’ kiwifruit (HY) is an early maturing variety with a fruit ripening period from August to September, and it has red flesh. ‘Jinyan’ kiwifruit (JY) is a late maturing variety with a fruit ripening period from October to November, and it has yellow flesh. The morphology of the two varieties’ leaf litters is shown in Figure 1. Due to differences in variety and planting area, there were significant differences in the initial nutrient contents of the litters and soil fungal communities between HY and JY (Table 1). The initial contents of C and N, and the values of C/N and C/P, were higher in the HY litter. The initial contents of P and K were higher in the JY litter. The relative abundances of *Lophotrichus* and *Botryotrichum* were higher in the initial soil of the HY orchard. The relative abundances of *Humicola*, *Tausonia*, and *Apiotrichum* were higher in the initial soil of the JY orchard.

### 2.2. Litter Decomposition Experiment

In the orchard, the kiwifruit trees were planted in a 2 m × 4 m area, and the average number of trees was 825/hm^2^. The annual fertilization amount of each hectare orchard was 4.5 t of organic fertilizer, 150 kg of urea, 300 kg of Ca(H_2_PO_4_)_2_, and 150 kg of K_2_SO_4_. The fertilization times were February, May, and November, and each fertilizer amount accounted for 20%, 30%, and 50% of the annual fertilizer amount, respectively. Irrigation depended on the precipitation situation, and a soil field capacity of over 60% was maintained. In December 2021, branches and leaves of kiwifruit were pruned and covered on the soil surface of the orchard. In the planting areas of HY and JY, five quadrats of 5 m × 5 m in size were randomly established, respectively. The leaf litter was collected and taken back to the laboratory in each quadrat, and then the litter sample was dried for 48 h at 60 °C for preservation. The litter bag method was used in the litter decomposition experiment. A nylon mesh bag of 15 cm × 25 cm in size with a 0.5 mm mesh diameter was used as the litter decomposition bag. A total of 10.0 g of dried litter samples was weighed and placed into the litter bag. A total of 30 litter bags were prepared for HY and JY, respectively. A portion of litter samples was reserved for the determination of the initial nutrient content.

The litter decomposition experiment was performed in the kiwifruit orchard. In June 2022, the litter bags were laid flat and placed on the soil surface in the quadrat. Five bags were placed in one quadrat and secured with nails. There were a total of 5 quadrats and 30 litter bags in each planting area of HY and JY, respectively. A litter bag was retrieved and taken back to the laboratory every 15 days in each quadrat. The litter samples were dried for 48 h at 60 °C after removing debris such as soil and plant roots, and then they were stored to determine the mass and nutrient content of the residual samples. At the beginning of the decomposition experiment and each time the litter bag was retrieved, 50 g of the surface soil under the litter bag was taken in each quadrat. After removing sand, rocks, plant roots, and other debris, the soil sample was stored at −80 °C to determine the microbial community. From 15 June to 12 September 2022, the litter decomposition experiment was carried out 90 days with a total of six samples. Based on our previous research, the time taken for kiwifruit litter to decompose by 50% was 40 to 70 days [28]. Considering the number of samples required for this experiment, the decomposition period was set to 90 days.

The mass of residual litter sample was measured by electronic balance. The nutrient contents were determined according to the methods used by Lu et al. [28]. The contents of C and N were determined using an element analyzer (vario MACRO cube, Germany Elementar, Langenselbold, Germany). The content of P was determined using an ultraviolet spectrophotometer (UV-1800, Japan Shimadzu, Kyoto, Japan) based on the colorimetric method. The content of K was determined using an atomic absorption spectrometer (Analyst 800, USA PE, Cincinnati, OH, USA).

### 2.3. Soil Microbial Community Analysis

The total genomic DNA of soil samples was extracted using the OMEGA Soil DNA Kit (Omega Bio-Tek, Norcross, GA, USA) and then stored at −20 °C. The quantity of extracted DNA was determined using a NanoDrop NC2000 spectrophotometer (Thermo Fisher Scientific, Waltham, MA, USA), and the quality was determined using agarose gel electrophoresis. For fungi, PCR amplification of ITS rRNA genes in the V1 region was performed using the forward primer ITS5-1737F (5′-GGAAGTAAAAGTCGTAACAAGG-3′) and the reverse primer ITS2-2043R (5′-GCTGCGTTCTTCATCGATGC-3′). For bacteria, PCR amplification of 16S rRNA genes in the V3-V4 regions was performed using the forward primer 338F (5′-ACTCCTACGGGAGGCAGCA-3′) and the reverse primer 806R (5′-GGACTACHVGGGTWTCTAAT-3′). The barcode in the primer was a 7-base oligonucleotide sequence used to distinguish different samples within the same library. The PCR reaction system contained 0.25 μL of Q5 high-fidelity DNA polymerase, 5 μL of Reaction Buffer, 5 μL of High GC Buffer, 2 μL of dNTP, 2 μL of DNA template, 1 μL of forward primer, 1 μL of reverse primer, and 8.75 μL of ddH_2_O. The reaction conditions for PCR amplification consisted of pre-denaturation at 98 °C for 30 s, followed by 25 cycles consisting of denaturation at 98 °C for 15 s, annealing at 50 °C for 30 s, and extension at 72 °C for 30 s. Finally, extension was performed at 72 °C for another 5 min, and the samples were stored at 4 °C. PCR products were purified with Vazyme VAHTSTM DNA Clean Beads (Vazyme, Nanjing, China). Quantification was performed by using a Quant-iT PicoGreen dsDNA Assay Kit (Invitrogen, Carlsbad, CA, USA). After quantification, the Illumina NovaSeq 6000 platform with a NovaSeq 6000 SP Reagent Kit (Illumina, San Diego, CA, USA) (500 cycles) was used for pair-end 2 × 250 bp sequencing.

Microbiome bioinformatics was performed with Quantitative Insights Into Microbial Ecology (QIIME2 2022.11). Raw sequence data were demultiplexed using the demux plugin, and then primers were cut with the cutadapt plugin. The DADA2 (QIIME2 2022.11) plugin was used to filter quality, denoise, merge, and remove chimera from the sequence. The obtained sequences were merged according to 100% sequence similarity, and the amplicon sequence variants (ASVs) and abundance data tables were generated. The taxonomic information of ASV was obtained by comparing the ASV feature sequence with the reference sequence in the database. The database of fungi was unite_8, and the database of bacteria was silva_132. Moreover, the ASVs whose abundance values were less than 0.001% of the total samples were removed. The above work, including DNA sequencing and ASV classification, was carried out with assistance from Shanghai Personal Biotechnology Co., Ltd. (Shanghai, China).

### 2.4. Data Analysis

Sequence data processing and analysis mainly used QIIME2 (2022.11) and R packages (3.3.0). Based on the ASV abundance table, the specific composition of the microbial communities in each sample at each taxonomic level was obtained. In this study, the composition of the microbial community at the phylum and generic levels was mainly analyzed. QIIME2 (2022.11) software was used to randomly sample the total number of sequences of each sample in the ASV abundance matrix at different depths, and sparse curves were drawn based on the number of sequences extracted at each depth and their corresponding ASV numbers so as to judge whether the current sequencing depth of each sample was sufficient to reflect the microbial diversity contained in the community sample. The ASV abundance matrix was randomly flattened with 95% of the sequence quantity of the sample with the lowest sequence quantity in all samples so as to correct the diversity difference between samples caused by sequencing depth. Chao1 and Shannon diversity indexes were calculated using QIIME2 to show the species diversities within the habitat or community (alpha diversity). The Chao1 index represents community richness and can reflect the actual number of species in the community. The Shannon index comprehensively considers the richness and evenness of communities and is more suitable for complex communities. The higher the values of the Chao1 and Shannon diversity indexes, the higher the community diversities. A principal coordinate analysis (PCoA) was conducted to show the species diversities between different habitats or communities (beta diversity). PCoA takes the sample distance as a whole and is more consistent with the characteristics of ecological data compared with a principal component analysis (PCA). The distance algorithm was Bray–Curtis distance, and the elliptic confidence was 0.95.

Analyses of composition differences and marker species between communities were mainly performed using R (3.3.0) and Python (3.4) packages. Venn diagram for the number of ASVs in samples was created based on the ASV abundance table. Species composition heat maps were used to illustrate the distribution trend of species abundance in the sample, and the top 10 phyla and genera in abundance were selected in this study. The data standardization method was row normalization, and the sample and species clustering algorithms were average algorithms.

Linear discriminant analysis effect size (LEfSe) was used to detect the biomarker at all taxonomic levels between communities. Firstly, the data were filtered by abundance, the filtering threshold was set to 0.05, and species with abundance values lower than 0.05% were removed. Then, a one-against-all comparison strategy was used to screen out the different species, and Wilcoxon’s test was used to test the significance of the differences. Finally, a linear discriminant analysis (LDA) was carried out on the different species, and those whose effect size of difference exceeded the LDA threshold were the marker species. LDA threshold was set to 3.5.

Based on the association analysis method, a network analysis was used to show the co-occurrence or co-exclusion relationships between microbial groups. The top 20 relative abundances of generic-level fungi and bacteria were selected as objects of microbial groups. The correlation coefficient was the Spearman correlation coefficient, and the threshold of similarity was set to 0.6. In addition, a redundancy analysis (RDA) was used to explain the response of microorganisms to litter nutrient content during decomposition. The top 10 relative abundances of generic-level fungi and bacteria were selected as microorganisms, and the contents of C, N, P, and K in litters were selected as litter nutrient contents. The sorting method was goodness of fit, and the elliptic confidence was 0.95.

## 3. Results

### 3.1. Microbial Community Structure

**Figure 2:**
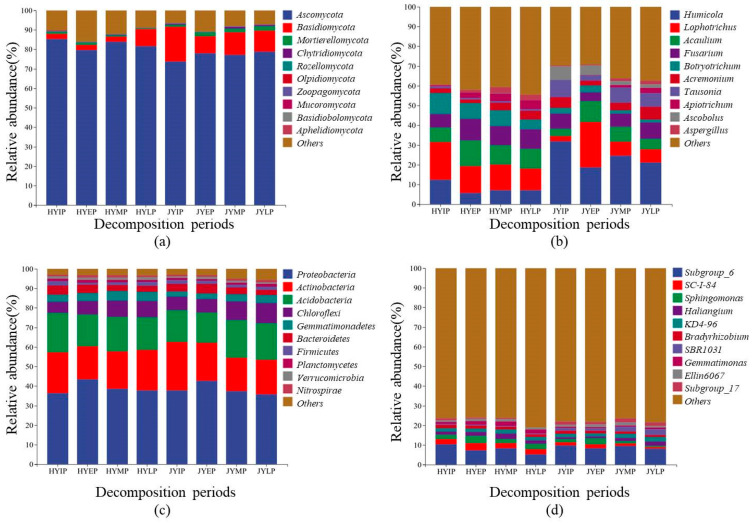
Microbial community compositions during kiwifruit litter decomposition. (**a**,**b**) show community compositions of fungi at phylum level and generic level, respectively. (**c**,**d**) show community compositions of bacteria at phylum level and generic level, respectively. HYIP, HYEP, HYMP, and HYLP represent microbial communities at initial, early, middle, and later periods of litter decomposition of ‘Hongyang’ kiwifruit. JYIP, JYEP, JYMP, and JYLP represent microbial communities at initial, early, middle, and later periods of litter decomposition of ‘Jinyan’ kiwifruit. Same in below figure.

Based on the litter decomposition pattern, combined with the weight loss rate of the kiwifruit litter, the decomposition experiment was divided into four periods. The start of the experiment was the initial period (IP), the first 30 days of the experiment was the early period (EP), 30 to 60 days was the middle period (MP), and 60 to 90 days was the later period (LP). The residue weights of the HY and JY litters during decomposition are shown in Figure S1; the JY litter had a faster weight loss rate. During the litter decomposition of HY and JY, the microbial community composition in the phylum was similar. *Ascomycota* and *Basidiomycota* were the dominant fungi, and their relative abundances were about 80% and 10%, respectively (Figure 2). *Proteobacteria*, *Actinobacteria*, and *Acidobacteria* were the dominant bacteria, and their relative abundances were about 40%, 20%, and 18%, respectively. However, the microbial community structure in the genera was different. For HY, *Lophotrichus*, *Acaulium*, *Fusarium*, *Humicola*, and *Botryotrichum* were the dominant fungi. Their relative abundances were about 13%, 11%, 10%, 7%, and 7%, respectively. *Subgroup_6*, *SC-I-84*, *Sphingomonas*, *Haliangium*, and *JG30-KF-AS9* were the dominant bacteria. Their relative abundances were about 7%, 3%, 3%, 2%, and 2%, respectively. For JY, *Humicola*, *Lophotrichus*, *Acaulium*, *Fusarium*, and *Tausonia* were the dominant fungi. Their relative abundances were about 22%, 12%, 8%, 6%, and 6%, respectively. *Subgroup_6*, *SBR1031*, *KD4-96*, *Subgroup_17*, and *Sphingomonas* were the dominant bacteria. Their relative abundances were about 9%, 2%, 2%, 2%, and 2%, respectively. The specific classifications of fungi and bacteria are shown in Table S2 and Table S3, respectively.

**Figure 3:**
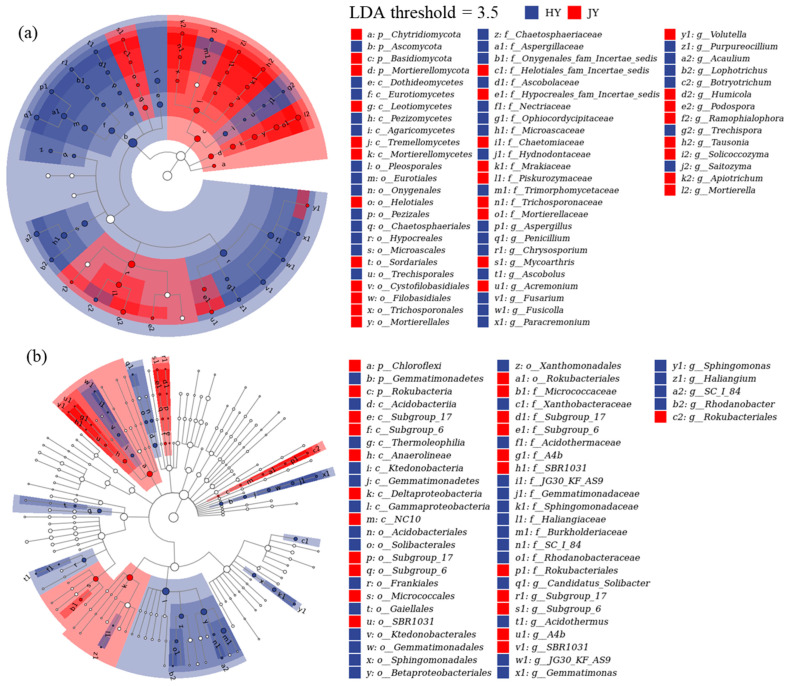
Dominant biomarkers of kiwifruit litters based on linear discriminant analysis effect size (LEfSe). (**a**,**b**) show LEfSe results of fungi and bacteria, respectively. HY represents ‘Hongyang’ kiwifruit, and JY represents ‘Jinyan’ kiwifruit. To enhance readability of this figure, legend only retains microorganisms at phylum and genus classification levels.

For the HY litter, the dominant biomarker fungi were *Ascobolus*, *Aspergillus*, *Penicillium*, *Acaulium*, *Lophotrichus*, and *Fusarium*, and the dominant biomarker bacteria were *Candidatus_Solibacter*, *JG30-KF-AS9*, *Acidothermus*, *Haliangium*, *SC-I-84*, *Sphingomonas*, and *Gemmatimonas* (Figure 3). For the JY litter, the dominant biomarker fungi were *Humicola* and *Tausonia*, and the dominant biomarker bacteria were *Subgroup_6*, *Subgroup_17*, and *SBR1031*.

### 3.2. Microbial Community Diversity

**Figure 4:**
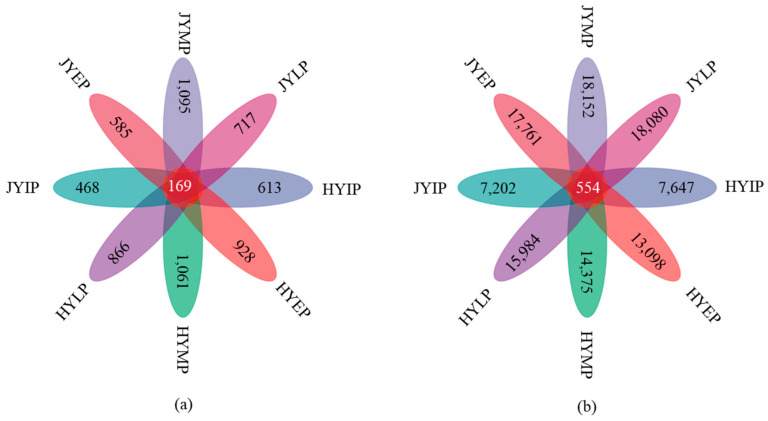
Venn diagram for number of ASVs during kiwifruit litter decomposition. (**a**) shows number of ASVs in fungi. (**b**) shows number of ASVs in bacteria.

In the whole decomposition period, the number of fungal ASVs in the HY litter was 4144, and the number of bacterial ASVs was 53,320 (Figure 4). The number of fungal ASVs in the JY litter was 3541, and the number of bacterial ASVs was 63,411. The number of fungal ASVs shared by both litters was 169, and the number of bacterial ASVs shared by both litters was 554. The number of fungal ASVs in the HY litter was higher, while the number of bacterial ASVs in the JY litter was higher. During litter decomposition, the number of fungal ASVs increased first and then decreased, and the number was the highest in the middle period of decomposition. The number of bacterial ASVs showed an increased trend, and the number was the highest in the later period.

**Figure 5:**
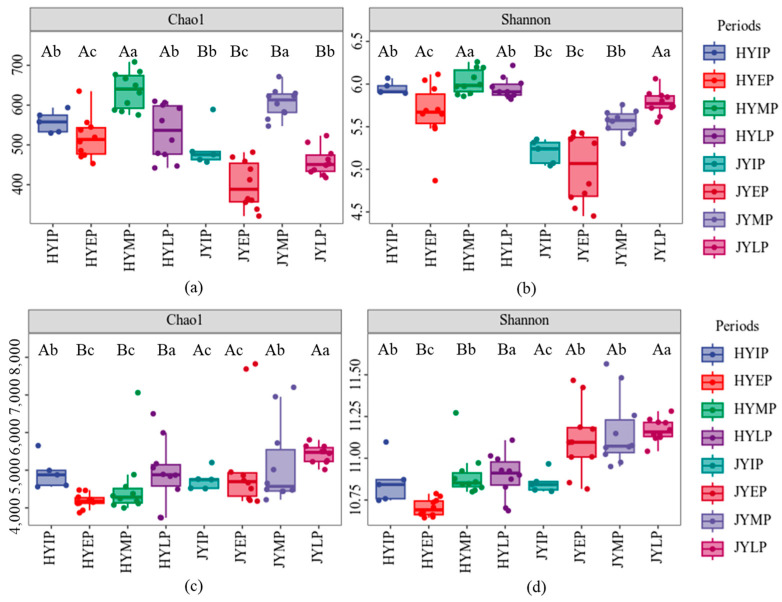
Microbial community diversity index during kiwifruit litter decomposition. (**a**,**b**) show Chao1 and Shannon diversity indexes of fungal community, respectively. (**c**,**d**) show Chao1 and Shannon diversity indexes of bacterial community, respectively. Different uppercase letters indicate significant differences in varieties, and different lowercase letters indicate significant differences in decomposition periods (*p* < 0.05).

Based on the Chao1 and Shannon diversity indexes, the fungal community diversity of HY was higher than that of JY during litter decomposition (Figure 5). The Chao1 indexes of HY were in the range of 518.52~636.97, while those of JY were in the range of 400.24~606.07. The Shannon indexes of HY were in the range of 5.66~6.03, while those of JY were in the range of 5.01~5.78. Moreover, the diversity indexes were higher in the middle and later periods. However, the bacterial community diversity of JY was higher. The Chao1 indexes of HY were in the range of 4183.46~4930.65, while those of JY were in the range of 5160.10~5424.04. The Shannon indexes of HY were in the range of 10.70~10.90, while those of JY were in the range of 11.11~11.17. Similarly to the fungal community, the diversity indexes were higher in the middle and later periods. After the input of the JY litter, there was a significant increase in bacterial diversity. The Chao1 index increased from 4749.93 to 5184.90, and the Shannon index increased from 10.86 to 11.11.

### 3.3. Microbial Community Succession

**Figure 6:**
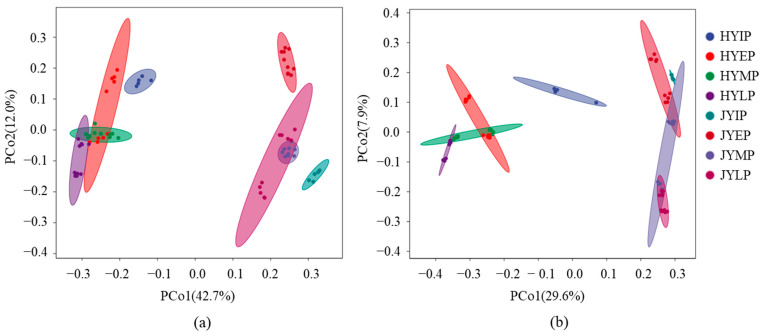
Microbial community succession based on PCoA (principal coordinate analysis) during kiwifruit litter decomposition. (**a**,**b**) show PCoA results of fungi and bacteria, respectively.

Based on the results of the PCoA (principal coordinate analysis), there were significant differences in the microbial community structure between the decomposition process of the HY and JY litters (Figure 6). Furthermore, the Bray–Curtis distance of the fungal community was greater than that of the bacterial community. There is a significant distance between the initial, early, and later periods of decomposition.

**Figure 7:**
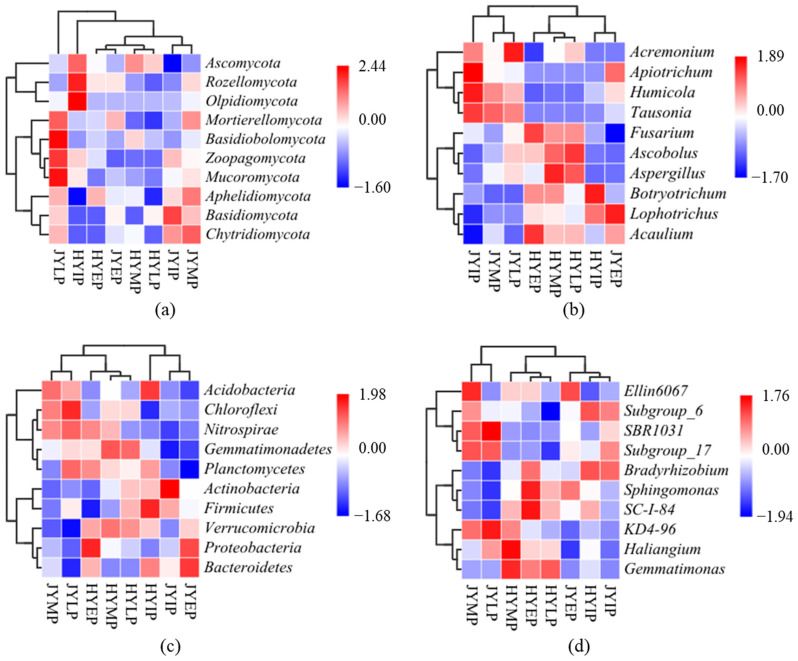
Microbial community succession based on composition heat maps during kiwifruit litter decomposition. (**a**,**b**) show composition heat maps of fungi at phylum and generic levels, respectively. (**c**,**d**) show composition heat maps of bacteria at phylum and generic levels, respectively.

Litter input significantly affected the soil microbial community structure. After the input of the HY litter, at the phylum level, the abundance of *Proteobacteria* increased, while the abundances of *Ascomycota*, *Acidobacteria*, and *Actinobacteria* decreased (Figure 7). At the generic level, the abundances of *Acaulium*, *Fusarium*, *Sphingomonas*, and *SC-I-84* increased, while the abundances of *Botryotrichum* and *Subgroup_6* decreased. After the input of the JY litter, at the phylum level, the abundances of *Proteobacteria* and *Bacteroidetes* increased, while the abundances of *Chytridiomycota*, *Basidiomycotain*, and *Actinobacteria* decreased. At the generic level, the abundances of *Acaulium*, *Lophotrichus*, *Ellin6067*, and *Sphingomonas* increased, while the abundances of *Humicola*, *Tausonia*, *Subgroup_6*, and *Bradyrhizobium* decreased.

At different periods of decomposition (early, middle, and later), the composition and abundance of the microbial community changes, which is known as microbial community succession. During the litter decomposition of HY, at the phylum level, the abundances of *Basidiomycota*, *Chloroflexi*, *Gemmatimonadetes*, and *Actinobacteria* increased, while the abundances of *Proteobacteria* and *Bacteroidetes* decreased. At the generic level, the abundance of *Ascobolus* increased, while the abundances of *Acaulium*, *Fusarium*, *Sphingomonas*, and *SC-I-84* decreased. During litter decomposition of JY, at the phylum level, the abundances of *Chytridiomycota*, *Acidobacteria*, and *Chloroflexi* increased, while the abundances of *Proteobacteria* and *Bacteroidetes* decreased increased. At the generic level, the abundances of *Acremonium*, *Haliangium*, and *KD4-96* increased, while the abundances of *Lophotrichu*, *Acaulium*, *Ellin6067*, and *Sphingomonas* decreased.

### 3.4. Effects of Biotic and Abiotic Factors on Microbial Communities

**Figure 8:**
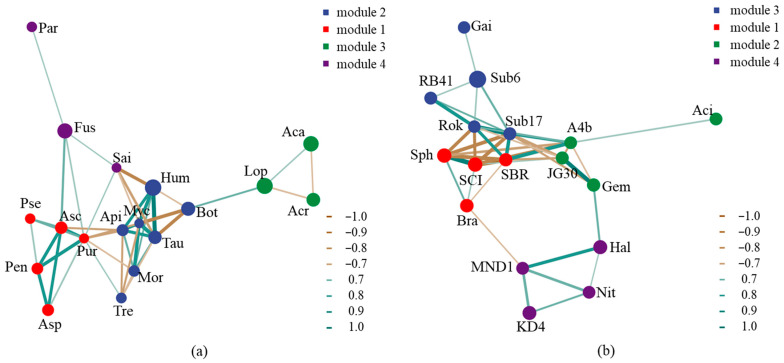
Co-occurrence and co-exclusion relationships between microorganisms based on network analysis. (**a**,**b**) show network analysis results of fungi and bacteria, respectively. Hum (*Humicola*), Lop (*Lophotrichus*), Aca (*Acaulium*), Fus (*Fusarium*), Bot (*Botryotrichum*), Acr (*Acremonium*), Tau (*Tausonia*), Asc (*Ascobolus*), Api (*Apiotrichum*), Asp (*Aspergillus*), Pen (*Penicillium*), Mor (*Mortierella*), Tre (*Trechispora*), Par (*Paraphaeosphaeria*), Pse (*Pseudogymnoascus*), Pur (*Purpureocillium*), Sai (*Saitozyma*), Myc (*Mycoarthris*); Sub6 (*Subgroup_6*), Sph (*Sphingomonas*), SCI (*SC-I-84*), Hal (*Haliangium*), KD4 (*KD4-96*), Bra(*Bradyrhizobium*), SBR (*SBR1031*), Gem (*Gemmatimonas*), Sub17 (*Subgroup_17*), RB41 (*RB41*), JG30 (*JG30-KF-AS9*), MND1 (*MND1*), Aci (*Acidibacter*), Nit (*Nitrospira*), A4b (*A4b*), Rok (*Rokubacteriales*), Gai (*Gaiella*).

Based on the results of the network analysis, there were co-occurrence and co-exclusion relationships between microbial groups during litter decomposition. Moreover, different microorganisms form specific modules to perform ecological functions. In fungi, *Humicola*, *Botryotrichum*, *Tausonia*, *Apiotrichum*, and *Mortierella* formed a function module with co-occurrence relationships (Figure 8). *Ascobolus*, *Aspergillus*, *Penicillium*, *Pseudogymnoascus*, and *Purpureocillium* formed a function module with co-occurrence relationships. In bacteria, *Subgroup_6*, *Subgroup_17*, *RB41*, *Rokubacteriales*, and *Gaiella* formed a function module with co-occurrence relationships. *Haliangium*, *KD4-96*, *MND1*, and *Nitrospira* formed a function module with co-occurrence relationships. In addition, there were co-exclusion relationships between *Sphingomonas* of *Proteobacteria* and *Subgroup_17* of *Acidobacteria*.

**Figure 9:**
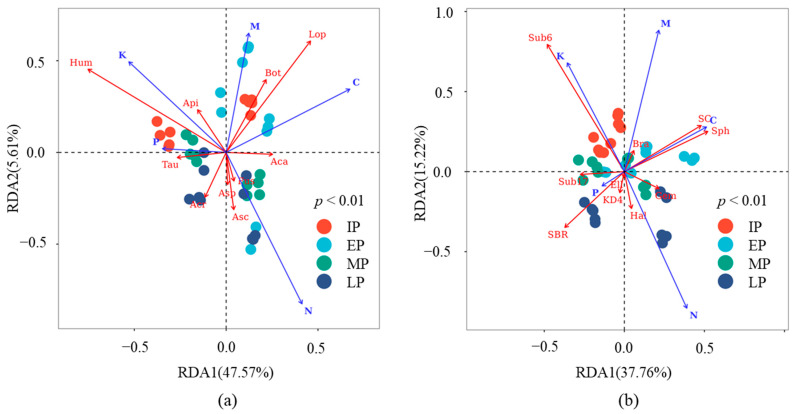
Response of microorganisms to litter nutrient content based on redundancy analysis (RDA). (**a**,**b**) show RDA results of fungi and bacteria, respectively. IP, EP, MP, and LP represent microbial communities at initial, early, middle, and later decomposition periods. Hum (*Humicola*), Lop (*Lophotrichus*), Aca (*Acaulium*), Fus (*Fusarium*), Bot (*Botryotrichum*), Acr (*Acremonium*), Tau (*Tausonia*), Asc (*Ascobolus*), Api (*Apiotrichum*), Asp (*Aspergillus*); Sub6 (*Subgroup_6*), Sph (*Sphingomonas*), SC (*SC-I-84*), Hal (*Haliangium*), KD4 (*KD4-96*), Bra(*Bradyrhizobium*), SBR (*SBR1031*), Gem (*Gemmatimonas*), Ell(*Ellin6067*), Sub17(*Subgroup_17*).

The nutrient contents in the litters explained 53.18% and 52.98% of the variations in generic abundances in the fungal and bacterial communities, respectively. The contents of C and N in the litters were the main factors affecting microbial communities (Figure 9). For fungi, the abundances of *Lophotrichus* and *Botryotrichum* were positively correlated with the content of C, and their abundances were higher in the early period of decomposition. The abundances of *Humicola* and *Apiotrichum* were negatively correlated with the contents of C and N. Therefore, *Humicola* and *Apiotrichum* were dominant fungi in the process of C and N degradation. The abundances of *Acremonium* and *Tausonia* were negatively correlated with the content of C, and their abundances were higher in the middle and later periods of decomposition. For bacteria, the abundances of *Sphingomonas* and *SC-I-84* were positively correlated with the content of C, and their abundances were higher in the early period of decomposition. The abundances of *SBR1031* and *Subgroup_17* were negatively correlated with the contents of C and N, and their abundances were higher in the middle and later periods of decomposition. The abundances of *Subgroup_6* were negatively correlated with the content of N. The nutrient content of kiwifruit litter during the decomposition process was shown in Table S4.

## 4. Discussion

Based on the results of this study, we verified the initial hypotheses. There were variety differences in the microbial communities corresponding to the decomposition processes of the HY and JY litters. These variety differences were reflected in the dominant microbial decomposers (community structure), community diversities, and community succession processes. At the generic level, *Lophotrichus*, *Acaulium*, and *Fusarium* were relatively more abundant in the microbial community of the HY litter, and *Humicola* and *Tausonia* were relatively more abundant in the microbial community of the JY litter. The bacterial community diversity of the JY litter was higher than that of the HY litter. The community diversity was higher in the middle and later periods. The mechanisms of variety differences were related to litter quality and the initial soil microbial community. The contents of C and N in the litters were the main factors affecting microbial communities.

### 4.1. Dominant Microbial Decomposer

At the phylum level, *Ascomycota* and *Basidiomycota* were the dominant fungal decomposers during kiwifruit litter decomposition, which is consistent with many previous studies. *Ascomycota* are decomposers of hemicellulose or cellulose, while *Basidiomycota* are decomposers of lignocellulose and can produce many lignocellulolytic enzymes [9]. *Basidiomycota* can degrade various types of substrates [29] and were more abundant during the decomposition of the JY litter. At the generic level, *Lophotrichus*, *Acaulium*, *Fusarium*, and *Humicola* were the dominant fungal decomposers. *Acaulium* can efficiently degrade easily available total sugars and cellulose and is also a genus of lignin-degrading fungi [30]. *Lophotrichus* is a dominant genus in *Ascomycota* [30], and it belongs to the same family *Microascaceae* with *Acaulium*. *Fusarium* participates in soil organic matter decomposition, N mineralization, and nitrification, producing peroxidase and degrading stubborn compounds such as lignocellulose [12,31]. *Humicola* participates in soil C and N cycling and denitrification [31]. Moreover, *Humicola* and *Tausonia* were the dominant biomarker fungi of the JY litter. *Humicola* can produce various lignocellulose-degrading enzymes, including thermally stable cellulases, hemicellulases, ligninases, amylases, and glucose isomerases. *Tausonia* is a genus of class *Tremellomycetes*, and they are able to degrade hemicellulose and assimilate phenolic compounds [32].

At the phylum level, *Proteobacteria*, *Actinobacteria*, and *Acidobacteria* were the dominant bacterial decomposers during kiwifruit litter decomposition. *Proteobacteria* are typical R-strategy bacteria that produce cellulase enzymes and decompose simple organic matter [33,34]. *Actinobacteria* are copiotrophs and R-strategy bacteria and can secrete glycoside hydrolases and lignolytic enzymes [33]. In contrast, *Acidobacteria* are oligotrophs and typical K-strategy bacteria [35] and are able to grow in lignocellulose and fungal chitin [36]. At the generic level, *Subgroup_6* and *Sphingomonas* were the dominant bacterial decomposers. As a genus of *Acidobacteria*, *Subgroup_6* participates in carbohydrate metabolism and transforms carbohydrates into monosaccharides [37]. With abundant denitrifying functional genes, *Sphingomonas* are able to produce a wide range of proteolytic and cellulolytic enzymes, and it can degrade various types of substrates, including soluble and complex organic matter [38,39]. Furthermore, *Chloroflexi* were the dominant biomarker bacteria of JY litter. *Chloroflexi* are able to degrade cellobiose in the early decomposition period [40].

### 4.2. Community Diversity

According to the alpha diversity index, the fungal diversity of the HY litter was higher, while the bacterial diversity of the JY litter was higher. Bacteria can utilize easily degradable carbohydrates, rapidly proliferate, increase community richness and diversity, and rapidly degrade organic matter [41]. The driving effect of bacteria on the degradation of labile compounds (dissolved organic carbon) has been confirmed [42]. Therefore, bacteria play a decisive role in the decomposition rate of litter, especially in the early period. This may be one of the reasons why the weight loss rate of the JY litter is higher than that of the HY litter.

After the input of the JY litter, the diversity of soil bacterial communities significantly increased. Compared with the HY litter, the JY litter had a higher quality with a lower C content, a higher P content, and lower ratio values of C/N and C/P. Bacteria generally adopt the R strategy in the utilization of nutrient resources and have a higher utilization efficiency of litters with a low C/N ratio [6]. Sauvadet et al. [13] reported that the impact of high-quality litter on soil bacterial communities was greater than that of low-quality litter, which was consistent with this study.

### 4.3. Community Succession Along with the Decomposition Period

Community succession is the change in community composition along with the decomposition process, including two key stages. One is the initial period to the early decomposition period, and the other is the early period to the late period of decomposition. After the input of the kiwifruit litter, the abundance of *Acaulium* increased in fungi, and the abundance of *Proteobacteria* (including *Sphingomonas*) increased in bacteria. The input of litter provides large amounts of available sources of C, N, and P for microorganisms. Especially in the early period of decomposition, simple substrates such as small molecular sugars, amino acids, and starches are rapidly utilized by microorganisms to increase their own biomass. Therefore, *Acaulium* increased biomass by utilizing easily available total sugars, and *Sphingomonas* increased biomass by utilizing soluble organic matter. The high abundance of these two genera in the early decomposition period was consistent with previous studies [30,33,34].

Along with the decomposition process, the litter matrix transitions from labile substances (small molecule sugar, cellulose, and hemicellulose) in the early period to recalcitrant substances (lignin) in the late period. Furthermore, as the energy source and growth environment of microorganisms, the change in litter substrate will inevitably affect the microbial community. In the late period, the abundances of *Basidiomycota* and *Chytridiomycota* were higher, and they were reported as fungal decomposers of refractory substrates [43]. At the generic level, there were significant differences in the dominant fungi during the middle and later periods between the HY and JY litters. The abundances of *Aspergillus* and *Ascobolus* were higher in the HY litter, while the abundances of *Acremonium* and *Tausonia* were higher in the JY litter. *Acremonium* and *Aspergillus* were reported to be able to degrade recalcitrant compounds [22,30]. For bacteria, the abundances of *Proteobacteria* were higher in the early period and then decreased along with decomposition processes. As R-strategy and eutrophic bacteria, *Proteobacteria* prefer to utilize unstable carbon sources and simple organic matter and are dominant in the early period of decomposition [36]. As genera of *Proteobacteria*, the abundances of *Sphingomonas*, *SC-I-84*, and *Ellin6067* decreased in the later period. Moreover, *Sphingomonas* dominating in the early stages of litter decomposition has been confirmed in many studies [33,34,44]. During the later period, the abundances of *Actinobacteria* and *Gemmatimonadetes* were higher in the HY litter, while the abundances of *Acidobacteria* were higher in the JY litter. Because of the higher weight loss and the higher content of stubborn substances in the JY litter during the later period, *Acidobacterium* dominated with an ability to utilize recalcitrant carbon substrates [33]. In addition, the phenomenon of *Actinobacteria* replacing *Proteobacteria* along with the decomposition process has been found in previous studies [14,34]. *Actinobacteria* are able to secrete a wider range of degrading enzymes to adapt to various substrates [36].

### 4.4. Litter Quality and Microbial Community

Litter quality is a critical factor affecting microbial community. The litter selects the microbial communities by substrate quality, and in turn, microbial communities convert the substrate quality by enzymatic hydrolyses [15]. The C:N:P stoichiometry reflects the litter quality and affects the microbial community structure [45]. Based on the concept of the threshold element ratio (TER), the value at which microbial growth switches from C to N limited or vice versa can reflect the relationship between the litter quality, decomposition rate, and microbial nutrient requirements [46]. Microorganisms drive C and N metabolism by keeping their TER matched to the litter substrate. The process of microbial C and N metabolism is as follows: first, lignocellulose, proteins, and lipids are degraded into polysaccharides, peptides, and fatty acids; then, they are further degraded into disaccharides and amino acids; and finally, they are completely oxidized or enzymatically decomposed in the cell to produce CO_2_, H_2_O, NH_4_^+^, and NO_3_^−^ [39]. Therefore, the levels of C and N in the litter substrate are important factors affecting microbial communities. Wang et al. [47] found that differences in the litter quality result in variations in the composition and structure of soil microbial communities. In this study, the contents of C and N in the litter significantly affected the community structure. The abundance of *Humicola* was negatively correlated with the contents of C and N in the litter. *Humicola* is a genus of saprophytic fungi and participates in C and N cycling. Furthermore, Duan et al. [48] found that *Chaetomiaceae* (family of *Humicola*) were sensitive to exogenous N input and played an important role in soil C sequestration. In addition, some fungi participate in lignin or hemicellulose degradation, such as *Acremonium* and *Tausonia*, and their abundances were negatively correlated with the C content in the litter. Bacteria have a relatively high N content and low C content, so they have a high demand for exogenous N [36]. As the most abundant genus during the decomposition of kiwifruit litter, *Subgroup_6* was negatively correlated with the N content in the litter. Moreover, the N content affected the abundances of various bacteria, including *Subgroup_17* of *Acidobacteria*, *Haliangium* of *Proteobacteria*, *SBR1031* of *Chloroflexi*, and *Gemmatimonas* of *Gemmatimonadetes*. The driving effect of N on the bacterial community has been reported in many studies [35,49]. Fierer et al. [50] found that the abundance of *Acidobacteria* was negatively associated with C availability. In this study, the abundance of *Subgroup_17* (*Acidobacteria*) was negatively correlated with the content of C in the litter.

Except for litter quality, site conditions also have an impact on microbial communities, and the impact is greater for fungal communities [51]. Zhang et al. [52] reported that fungal communities were more susceptible to locations, and the beta diversities of fungal communities are more different along with the change in environment. There were significant differences in the initial soil fungal communities between the HY and JY litters. Moreover, the sample distances of the fungal community between the HY and JY litters were greater than those of the bacterial community based on the results of the PcoA.

In addition, microorganisms are affected by other microorganisms, with both synergistic and competitive relationships present. Based on the degradation of refractory substrates, *Chaetomiaceae* (*Humicola* and *Botryotrichum*) and *Basidiomycota* (*Tausonia* and *Apiotrichum*) had a co-occurrence relationship. With the ability to produce lignolytic peroxidases, *Aspergillus* and *Penicillium* had a co-occurrence relationship [12]. Based on the degradation of cellulose, *Subgroup_17*, *RB41*, *Rokubacteriales*, and *Subgroup_6* had a co-occurrence relationship [36,37]. Based on different survival strategies, there is a competitive relationship between *Proteobacteria* (R-strategy) and *Acidobacteria* (K-strategy), such as *Sphingomonas* and *Subgroup_17*.

Besides soil microorganisms, the phyllosphere microorganism is also one of the decomposers of litter. The phyllosphere microorganism dominates in the early stages of decomposition but is rapidly replaced by bacterial groups that secrete proteases and cellulases [53]. Although soil microorganisms are the main force driving the decomposition of kiwifruit litter, the role of the phyllosphere microorganism cannot be ignored, and it is one of our future research topics. In addition, this study analyzed the effects of the litter nutrient content on microbial communities but did not analyze the effects of soil nutrients. It must be noted that the experimental setup ensured consistency in the initial nutrient content and management measures of the orchard soil between the two varieties. However, changes in soil nutrients during litter decomposition may affect microbial communities. Yang et al. [54] found that greater soil fertility could facilitate litter decomposition. Especially for litters with poor N substrate, N transfer between litters and soil occurs during decomposition, and it is more susceptible to soil fertility [55]. Therefore, it is important to clarify the dynamic interaction between litter decomposition, microbial community, and soil nutrients, which is a necessary basis for scientific soil management in orchards.

## 5. Conclusions

During the litter decomposition of HY and JY, *Ascomycota* and *Basidiomycota* were the dominant fungi, and *Proteobacteria*, *Actinobacteria*, and *Acidobacteria* were the dominant bacteria. The differences in the microbial community structure between HY and JY are more reflected at the generic level. *Lophotrichus*, *Acaulium*, and *Fusarium* were relatively more abundant in the microbial community of the HY litter, and *Humicola* and *Tausonia* were relatively more abundant in the microbial community of the JY litter. The bacterial community diversity of JY was higher than that of the HY litter. Along with the decomposition process, the abundances of *Basidiomycota* and *Actinobacteria* increased in the HY litter, and the abundances of *Chytridiomycota* and *Acidobacteria* increased in the JY litter. The variety differences in microbial community were related to litter quality, especially the contents of C and N.

Here, we propose the following suggestions to promote the litter decomposition of fruit trees. On the one hand, microbial agents that dominate litter decomposition, such as *Humicola*, *Lophotrichus*, and *Sphingomona*, could be added to the orchard soil. It must be emphasized that the added microbial agents could not cause fruit tree diseases. On the other hand, it can be pruned proper early, or combined with organic and nitrogen fertilizers after pruning. By increasing the contents of C and N in the litter and soil, microbial activity is enhanced. In addition, it is suggested to take corresponding orchard management measures considering the differences in fruit tree varieties and soil conditions. This study could provide a reference for management measures of decomposition of pruning leaves in kiwifruit and other orchard ecosystems.

## Figures and Tables

**Table 1:** Initial nutrient contents of kiwifruit litter and initial relative abundance of fungi in orchard soil.

Nutrient Contents in Litters	HY	JY	Relative Abundance of Fungi in Soil	HY	JY
C (mg/g)	395.4 ± 4.6 *	334.1 ± 3.1	*Humicola* (%)	12.53 ± 0.91	32.02 ± 2.37 **
N (mg/g)	20.0 ± 1.1 *	18.7 ± 0.6	*Lophotrichus* (%)	19.05 ± 0.49 **	2.63 ± 0.25
P (mg/g)	1.8 ± 0.1	2.5 ± 0.1 *	*Acaulium* (%)	7.29 ± 1.19	3.68 ± 0.62
K (mg/g)	11.1 ± 0.7	17.9 ± 0.5 *	*Fusarium* (%)	6.82 ± 0.75	7.72 ± 0.46
C/N	19.44 ± 1.16	18.11 ± 0.72	*Botryotrichum* (%)	10.70 ± 1.17 *	2.77 ± 1.44
C/P	217.50 ± 8.24 **	133.75 ± 4.59	*Acremonium* (%)	2.45 ± 0.78	5.41 ± 0.80
C/K	35.56 ± 2.81 **	19.08 ± 0.66	*Tausonia* (%)	0.97 ± 0.16	8.74 ± 0.64 **
N/P	11.22 ± 0.81	7.39 ± 0.15	*Apiotrichum* (%)	0.02 ± 0.01	6.77 ± 0.65 **

HY represents ‘Hongyang’ kiwifruit, and JY represents ‘Jinyan’ kiwifruit. * *p* < 0.05; ** *p* < 0.01.

## Data Availability

The original contributions presented in the study are included in the article/Supplementary Material, further inquiries can be directed to the corresponding author.

## Supplementary Materials

The following supporting information can be downloaded at https://www.mdpi.com/article/10.3390/microorganisms12122498/s1, Figure S1: Residue weight of kiwifruit litter during decomposition. Table S1: Temperature and precipitation of study area during decomposition experiment. Table S2: Specific classification of dominant fungi. Table S3: Specific classification of dominant bacteria. Table S4: Nutrient content of kiwifruit litter during decomposition process.
