# Supplementary material for: Microbial Community Structure, Diversity, and Succession During Decomposition of Kiwifruit Litters with Different Qualities
Source: Microorganisms. 2024 Dec 4;12(12):2498. doi: 10.3390/microorganisms12122498 (PMC11727838; doi:10.3390/microorganisms12122498)
Supplement: Supplementary file 1 [file microorganisms-12-02498-s001.zip › Supplementary Material.pdf]

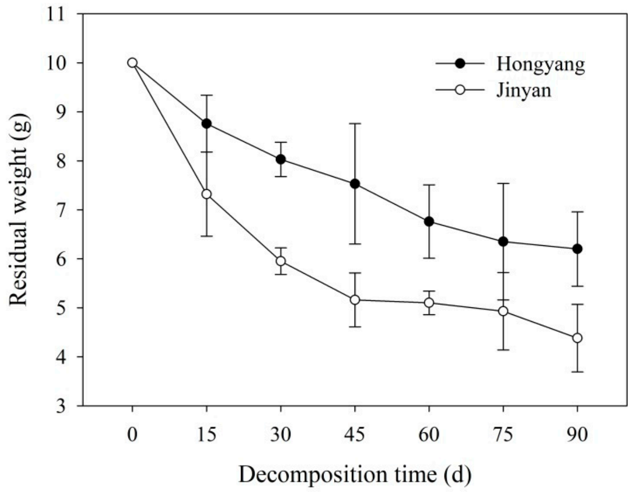

Figure S1 Residue weight of kiwifruit litter during decomposition

Table S1 Temperature and precipitation of study area during the decomposition experiment

Decomposition time (d)	Daily mean temperature (℃)	Daily precipitation (mm)
1	26	0
2	25.5	0
3	25.5	6.15
4	24	127.97
5	24	154.51
6	25.5	28.87
7	26	3.08
8	27.5	36.73
9	29	0
10	29	0
11	31	0
12	30.5	0
13	30	0
14	28.5	0
15	25.5	1.73
16	27	59.12
17	29	0
18	28.5	4.67
19	29	2.19
20	28.5	55.56
21	26.5	120.9
22	29	18.65
23	29	13.52
24	29.5	0
25	30	0
26	30.5	0

27	31	0
28	31.5	0
29	31	0
30	31.5	0
31	31.5	0
32	31.5	0
33	30	31.28
34	28.5	9.8
35	28.5	93.73
36	31	0
37	31.5	0
38	31.5	0
39	31.5	0
40	31.5	0
41	31.5	0
42	32	0
43	32.5	0
44	32.5	0
45	30.5	35.74
46	28.5	0
47	29.5	0
48	30.5	0.05
49	31	0
50	31	0
51	31	0
52	31.5	0
53	31	0
54	31.5	0
55	31.5	0
56	32	0
57	31.5	0
58	31.5	0
59	31.5	0
60	31.5	0
61	32	0
62	32	0
63	32.5	1.28
64	32.5	0
65	32.5	0
66	33	0
67	32.5	0
68	32.5	0
69	32.5	0
70	33	0

71	32	0
72	32	0
73	31.5	0
74	31.5	0
75	31	0.18
76	32	18.73
77	27.5	0
78	26	0
79	24.5	0
80	26	0
81	25.5	0
82	25.5	0
83	27.5	0
84	27	0
85	28.5	0
86	29	0
87	29	0
88	27.5	0
89	27.5	0
90	27.5	0

Table S2 Specific classification of dominant fungi

Phylum	Class	Order	Family	Genus
Ascomycota	Sordariomycetes	Microascales	Microascaceae	Lophotrichus
Ascomycota	Sordariomycetes	Microascales	Microascaceae	Acaulium
Ascomycota	Sordariomycetes	Sordariales	Chaetomiaceae	Humicola
Ascomycota	Sordariomycetes	Sordariales	Chaetomiaceae	Botryotrichum
Ascomycota	Sordariomycetes	Hypocreales	Nectriaceae	Fusarium
Ascomycota	Sordariomycetes	Hypocreales	Incertaesedis	Acremonium
Ascomycota	Sordariomycetes	Hypocreales	Ophiocordycipitaceae	Purpureocillium
Ascomycota	Eurotiomycetes	Eurotiales	Aspergillaceae	Aspergillus
Ascomycota	Eurotiomycetes	Eurotiales	Aspergillaceae	Penicillium
Ascomycota	Pezizomycetes	Pezizales	Ascobolaceae	Ascobolus
Ascomycota	Leotiomycetes	Thelebolales	Pseudeurotiaceae	Pseudogymnoascus
Ascomycota	Leotiomycetes	Thelebolales	Pseudeurotiaceae	Pseudeurotium
Ascomycota	Dothideomycetes	Pleosporales	Didymosphaeriaceae	Paraphaeosphaeria
Basidiomycota	Tremellomycetes	Cystofilobasidiales	Mrakiaceae	Tausonia
Basidiomycota	Tremellomycetes	Trichosporonales	Trichosporonaceae	Apiotrichum
Mortierellomycota	Mortierellomycetes	Mortierellales	Mortierellaceae	Mortierella

Table S3 Specific classification of dominant bacteria

Phylum	Class	Order	Family	Genus
Acidobacteria	Subgroup_6	Subgroup_6	Subgroup_6	Subgroup_6
Acidobacteria	Subgroup_17	Subgroup_17	Subgroup_17	Subgroup_17

Acidobacteria	Acidobacteriia	Solibacterales	Solibacteraceae	Candidatus_Solibacter
Acidobacteria	Blastocatellia	Pyrinomonadales	Pyrinomonadaceae	RB41
Proteobacteria	Gammaproteobacteria	Betaproteobacteriales	SC-I-84	SC-I-84
Proteobacteria	Alphaproteobacteria	Sphingomonadales	Sphingomonadaceae	Sphingomonas
Proteobacteria	Deltaproteobacteria	Myxococcales	Haliangiaceae	Haliangium
Proteobacteria	Alphaproteobacteria	Rhizobiales	Xanthobacteraceae	Bradyrhizobium
Proteobacteria	Gammaproteobacteria	Betaproteobacteriales	Nitrosomonadaceae	Ellin6067
Proteobacteria	Gammaproteobacteria	Betaproteobacteriales	Nitrosomonadaceae	MND1
Proteobacteria	Gammaproteobacteria	Incertae_Sedis	Unknown	Acidibacter
Chloroflexi	KD4-96	KD4-96	KD4-96	KD4-96
Chloroflexi	Anaerolineae	SBR1031	SBR1031	SBR1031
Chloroflexi	Ktedonobacteria	Ktedonobacterales	JG30-KF-AS9	JG30-KF-AS9
Chloroflexi	Anaerolineae	SBR1031	A4b	A4b
Actinobacteria	Actinobacteria	Frankiales	Acidothermaceae	Acidothermus
Actinobacteria	Thermoleophilia	Gaiellales	Gaiellaceae	Gaiella
Gemmatimonadetes	Gemmatimonadetes	Gemmatimonadales	Gemmatimonadaceae	Gemmatimonas
Rokubacteria	NC10	Rokubacteriales	Rokubacteriales	Rokubacteriales
Nitrospirae	Nitrospira	Nitrospirales	Nitrospiraceae	Nitrospira

Table S4 Nutrient content of kiwifruit litter during decomposition process

Nutrient	C(mg/g)		N(mg/g)		P(mg/g)		K(mg/g)	
Decomposition time (d)	Hongyang	Jinyan	Hongyang	Jinyan	Hongyang	Jinyan	Hongyang	Jinyan
0	395.4±10.2	334.1±15.4	20.0±1.5	18.7±2.3	1.8±0.3	2.5±0.5	11.1±1.3	17.9±1.8
15	406.7±15.3	423.4±20.6	22.3±2.2	21.9±1.6	1.7±0.2	2.4±0.6	3.3±0.6	1.6±0.5
30	408.4±14.9	384.9±16.7	26.9±3.1	24.3±2.9	2.2±0.6	2.2±0.5	1.3±0.2	1.6±0.4
45	358.9±18.9	442.2±22.5	22.7±2.8	24.0±2.4	2.5±0.5	2.1±0.4	2.2±0.4	1.5±0.3
60	388.0±11.5	342.7±17.5	26.0±3.5	25.7±3.1	0.9±0.2	2.0±0.3	0.7±0.1	1.4±0.2
75	394.9±23.2	312.1±12.6	28.0±3.1	27.0±3.6	0.4±0.1	2.1±0.2	1.6±0.3	1.7±0.2
90	372.6±20.5	363.1±13.8	24.4±2.8	24.2±2.7	4.0±0.6	2.1±0.5	3.6±0.8	1.8±0.3
